# Association between higher expression of interleukin-8 (IL-8) and haplotype −353A/−251A/+678T of *IL-8* gene with preeclampsia: A case–control study

**DOI:** 10.1097/MD.0000000000005537

**Published:** 2016-12-30

**Authors:** Lei Sun, Dongwei Mao, Yan Cai, Wenhua Tan, Yanlan Hao, Lin Li, Wei Liu

**Affiliations:** a Department of Gynaecology and Obstetrics, The Fourth Affiliated Hospital of Harbin Medical University; b Department of Gynaecology and Obstetrics, The Second Affiliated Hospital of Harbin Medical University, Harbin, China.

**Keywords:** IL-8, inflammation, preeclampsia, single nucleotide polymorphism

## Abstract

Preeclampsia (PE) is a common pregnancy-specific disorder associated with significant maternal and fetal morbidity and mortality worldwide.

The present study was performed to investigate the role of a CXC chemokine interleukin-8 (IL-8), in the pathogenesis of PE. IL-8 expression levels were assessed in placental and serum samples from 160 pregnant women with PE (N = 68 severe, 92 mild) and 140 healthy donors.

Results from enzyme-linked immunosorbent assay showed that the concentration of serum IL-8 in PE patients (180.27 ± 5.81 ng/L) was significantly higher than that in healthy controls (41.57 ± 5.67 ng/L). Patients with severe PE had even higher serum IL-8 levels. Similar messenger RNA and protein expression patterns of IL-8 in placental tissues were confirmed by quantitative real-time polymerase chain reaction and immunohistochemical assay (N = 30 each in the mild PE, severe PE, and control groups). In addition, single nucleotide polymorphisms of *IL-8* gene were detected with polymerase chain reaction-restricted fragment length polymorphism/SSP. The frequency of *IL-8*-251A allele was significantly higher than that in controls (58.4% vs 48.9%, *P* < 0.05). The occurrence frequency of haplotype −353A/−251A/+678T (AAT) in PE subjects was 27.2% as compared to 21.9% in the control participants (*P* < 0.05).

Our study reveals that IL-8 expression is positively associated with the severity of PE. Presence of haplotype AAT in pregnant women appears to be a risk factor for PE.

## 1 Introduction

Preeclampsia (PE) is a severe pregnancy-related disorder characterized by hypertension, proteinuria, and multiple organ damage.^[1]^ This disease is difficult to treat except by an early delivery, which may result in fetal complications.^[2]^ Several potential serum biomarkers for PE have been identified, such as soluble fms-like tyrosine kinase-1, placental growth factor, and soluble endoglin.^[3]^ Identification of these markers may help in early diagnosis, prevention, and treatment of this disease. Nonetheless, none of these have been proved to be of clinical value as yet.

Besides the impaired trophoblast invasion and aberrant vascular endothelial cell activation, accumulating evidence has shown that the development of PE is associated with exaggerated maternal inflammation.^[4,5]^ Activated neutrophils, monocytes, and natural killer cells initiate inflammation, which results in endothelial dysfunction.^[6]^ Interleukin-8 (IL-8), a member of the CXC family of chemokines, was originally discovered as the main chemoattractant for neutrophils; it has recently been found to be associated with several systemic inflammatory diseases, including PE.^[7]^ IL-8 is increased in PE subjects when compared with that in healthy controls, as demonstrated by Sharma et al in India^[8]^ and by Sahin et al in Turkey.^[9]^ However, only 54 and 41 PE patients were recruited in these 2 studies, respectively. To demonstrate the correlation between IL-8 expression and PE development, detection of its expression in a larger population is needed.

*IL-8* gene is located in chromosome 4q12-q21 and consists of 4 exons and 3 introns, which encode for a messenger RNA (mRNA) of 1605 bp. Polymorphisms of the *IL-8* gene have been implicated in several human diseases. For instance, the +781C/T and −352A/T polymorphisms of *IL-8* gene are related to cancer development,^[10,11]^ while the 4073A/T polymorphism is associated with myocardial infarction.^[12]^ Interestingly, increasing evidence has suggested that polymorphisms of inflammation-related genes may be associated with PE development, such as tumor necrosis factor-α, IL-10, and IL1A.^[13,14]^ However, whether the genetic variations in IL-8 are associated with the risk of PE is poorly studied.

In order to understand the role of IL-8 in the development and progress of PE in China, we tested the expression levels of IL-8 in serum and placental tissue samples from 160 PE subjects and 140 healthy pregnant women in the present study. Furthermore, potential single nucleotide polymorphisms (SNPs) of *IL-8* gene in the study population were detected. We found a direct correlation between IL-8 expression and the severity of PE, and that pregnant women with −353A/−251A/+678T (AAT) haplotype may be more susceptible to PE.

## 2 Materials and methods

### 2.1 Patients and healthy controls

The disease group included 160 PE patients registered between January 2010 and December 2013 at the Fourth Affiliated Hospital of Harbin Medical University, where they underwent obstetric examination and had a hospital delivery. Inclusion criteria for the control group were as follows: single pregnancy; Cesarean section termination of pregnancy; same types of anesthesia; no previous medical history of hypertension, heart disease, kidney disease, diabetes, hyperthyroidism, or other complications that may lead to vascular disorders and hypoxic changes; and no acute or chronic infectious diseases. A total of 140 healthy pregnant women who qualified the above-mentioned criteria in their third trimester were enrolled in this study. Written informed consent was obtained from all participants. The study protocol was approved by the Ethics Committee at the Harbin Medical University.

The diagnosis of “mild” and “severe” PE was determined according to the PE classification defined by American College of Obstetricians and Gynecologists. Patients with systolic blood pressure (SBP) >140 mm Hg, diastolic blood pressure (DBP) ≥90 mm Hg, and proteinuria >(+) were classified into mild PE (M-PE) group. Patients with ≥1 of the following were classified into the severe PE (S-PE) group: persistent high blood pressure, SBP ≥160 mm Hg and/or DBP ≥110 mm Hg; proteinuria ≥2.0 g/24 h or >(++); serum creatinine ≥1.2 mg/dL, except in patients in whom serum creatinine was increased before the test; platelets <100,000/mL (<100 × 10^9^/L); increased lactate dehydrogenase or evidence of microangiopathic hemolysis; elevated serum alanine/aspartate transaminase levels; persistent headache or other brain or visual impairment; and persistent upper abdominal pain. Blood pressure, gestational age, and urine protein were assessed to screen PE. Placental tissue samples from healthy controls and patients were rinsed with saline, and stored at −80°C until further use. Venous blood samples of the pregnant women in both groups were collected in 4-mL tubes coated with ethylenediaminetetraacetic (EDTA-K_2_, EDTA dipotassium salt), kept at room temperature for 30 minutes, and then centrifuged at 2000 to 3000 rpm for 10 minutes to collect serum. The extracted serum samples were also stored at −80°C until use.

### 2.2 Enzyme-linked immunosorbent assay

Serum IL-8 concentrations were determined using an enzyme-linked immunosorbent assay kit (R&D Systems Inc, Minneapolis, MN) according to the manufacturer's instructions, and expressed as nanogram per liter serum.

### 2.3 Quantitative real-time polymerase chain reaction

Total RNA was extracted from 0.5 cm^3^ placental tissue samples using TRIzol method (Invitrogen, Cambridge, MA). First-strand complementary DNA was synthesized from 2.0 μg total RNA using a First Strand Synthesis Kit (Thermo Fisher Scientific, Waltham, MA). A pair of primers (forward, 5′-CTTGGCAGCCTTCCTGATTT-3′; reverse, 5′-AACCCTCTGCACCCAGTTTT-3′) was designed to amplify a 238-bp region of *IL-8* gene (gene ID: 3576). Polymerase chain reaction (PCR) was performed on ABI StepOne plus real-time PCR system using SYBR Green Realtime PCR Master Mix (Toyobo Co Ltd, Osaka, Japan). β-Actin (*ACTB*) was used as a control (forward, 5′-TCAAGATCATTGCTCCTCCTG-3′; reverse, 5′-CTGCTTGCTGATCCACATCTG-3′; amplified products: 101 bp). mRNA expression levels of IL-8 were calculated using the 2^−ΔΔCT^ method.

### 2.4 Immunohistochemical assay

Placental tissue samples were cut into 1.0-cm^3^ cubes and fixed in neutral formalin for 48 hours. After dehydration, samples were paraffin embedded and cut into 4-μm thick slices. These slices were incubated with sodium citrate buffer for antigen retrieval, and subsequently with methanol–H_2_O_2_ solution (3%) to inactivate the endogenous peroxidase. Thereafter, the sections were incubated with 10% normal goat serum at 25°C for 3 hours, and then with anti–IL-8 antibody (1:200 dilution, ImmunoWay Biotechnology Company, Plano, TX) at 37°C for additional 2 hours. After being washed with phosphate-buffered saline, these slices were incubated with horseradish peroxidase–conjugated immunoglobulin G at 37°C for 30 minutes, treated with diaminobenzidine (KeyGen Biotech, Nanjing, China) for 3 to 8 minutes, and then stained with hematoxylin. Finally, these tissue slices were dehydrated with alcohol and xylene, sealed with neutral gum, and images obtained under a light microscope.

For analysis of IL-8 expression in placental specimens, 2 scoring rules were applied. Based on the ratio of IL-8–positive cells to total cells, specimens with 11% to 50% positive cells scored 2, 51% to 80% scored 3, and >81% scored 4. Additionally, based on the IL-8 expression intensity, weak, moderate, and strong staining was awarded a score of 1, 2, and 3, respectively. The placental specimens were then divided into 3 groups: negative (−), where the positivity rate of cells was <10% regardless of the staining intensity; weakly positive (+), where the combined score was between 3 and 5; or strongly positive (++) where the combined score was between 6 and 7.

### 2.5 Genotyping

Fasting venous blood samples were collected for genotyping. Genomic DNA was extracted by using a Blood Genomic DNA Extraction Kit (Takara, Japan) and stored at −20°C. *IL-8* genotypes −353A/T, −251T/A, and +678T/C were determined via PCR-restricted fragment length polymorphism (SSP) (gene ID: 3576). Sequence information of primer pairs is presented in Table 1.

**Table 1:**
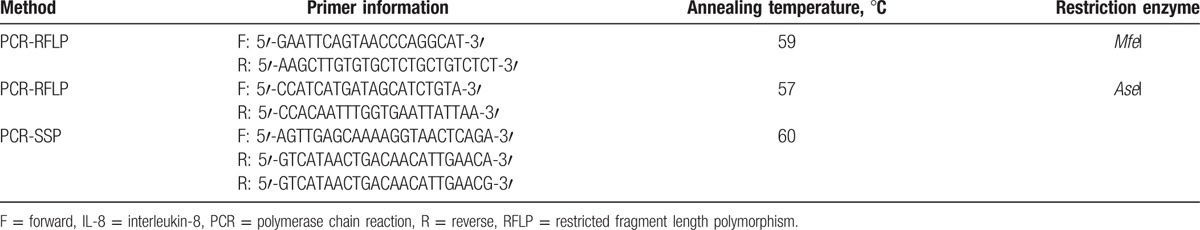
Primer and PCR reaction conditions for genotyping *IL-8* polymorphisms.

### 2.6 Statistical analysis

Data were analyzed using SPSS 19.0 (SPSS Inc, Chicago, IL). They are expressed as mean ± standard deviation or standard error. Between-group differences were assessed for statistical significance using unpaired *t* test or Mann–Whitney *U* test, as applicable. *P* < 0.05 was considered as significant. Haplotypes and their frequencies were estimated based on a Bayesian algorithm using the PHASE program. Chi-square test was carried out to analyze genotype, allele frequencies, and haplotype distribution of Hardy–Weinberg equilibrium. Nonconditional logistic regression model was used to calculate the odds ratio and 95% confidence intervals.

## 3 Results

### 3.1 Clinical characteristics

According to the American College of Obstetricians and Gynecologists criteria, a total of 92 patients were classified as M-PE, and the rest 68 were classified as S-PE. The baseline clinical characteristics of 140 healthy controls and 160 PE patients are summarized in Table 2. The gestational period of S-PE patients was 30.12 ± 1.33 weeks, which was significantly shorter than that in both M-PE patients (34.32 ± 1.52 weeks) and healthy controls (39.45 ± 1.50 weeks). Furthermore, both SBP and DBP were significantly higher in the 2 PE groups. Proteinuria was detected in M-PE (765.61 ± 132.81 mg) and S-PE (2865.56 ± 252.39 mg) patients, but not in controls.

**Table 2:**
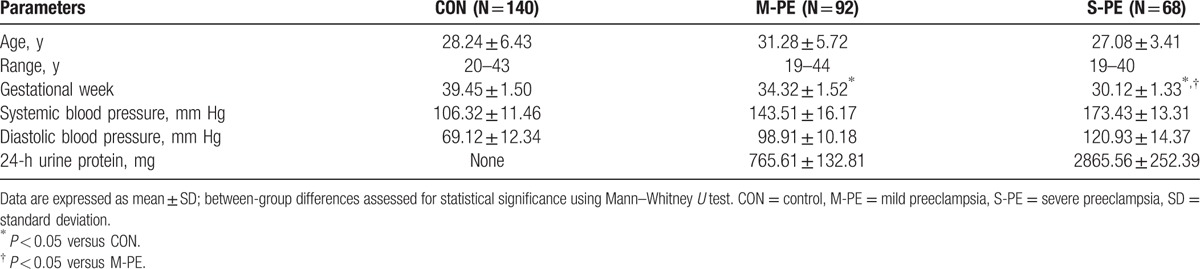
Baseline clinical characteristics of subjects.

### 3.2 IL-8 concentration is higher in the serum of PE patients

**Figure 1:**
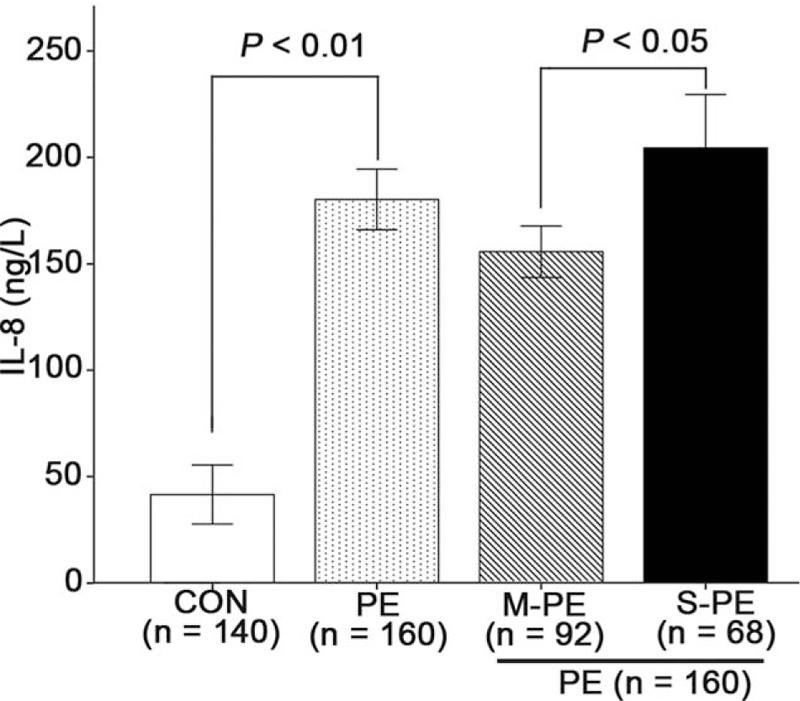
Higher serum IL-8 expression in PE patients. Serum IL-8 levels in venous blood in PE patients and healthy controls were determined by ELISA. Data presented as mean ± SD. Between-group differences were assessed for statistical significance using unpaired *t* test. CON = control, ELISA = enzyme-linked immunosorbent assay, IL-8 = interleukin-8, M-PE = mild preeclampsia, PE = preeclampsia, S-PE = severe preeclampsia, SD = standard deviation.

Serum IL-8 levels were determined in all participants using enzyme-linked immunosorbent assay. As indicated in Fig. 1, the mean IL-8 level in the PE group (180.27 ± 5.81 ng/L) was >4 times higher than that in the control group (41.56 ± 5.67 ng/L). Patients in the S-PE group had much higher serum IL-8 levels (204.53 ± 10.2 vs 155.73 ± 4.95 ng/L in the M-PE group).

### 3.3 Higher IL-8 expression in the placental tissues of PE patients

**Figure 2:**
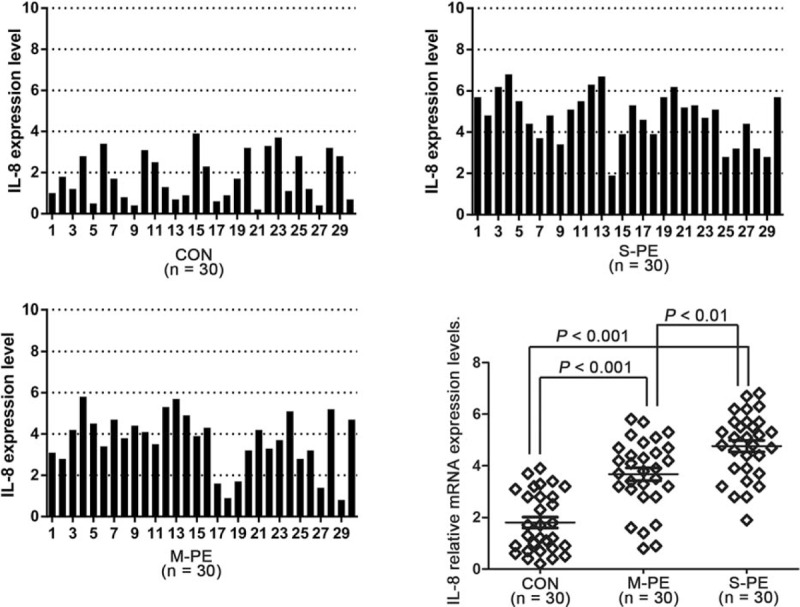
IL-8 mRNA expression is higher in placental tissues of PE subjects. The relative mRNA expression was determined in 30 samples randomly selected from each group. β-Actin was used as a control. Data presented as mean ± SD. Statistical significances between 2 groups were analyzed with unpaired *t* test. CON = control, IL-8 = interleukin-8, M-PE = mild preeclampsia, mRNA = messenger RNA, PE = preeclampsia, S-PE = severe preeclampsia, SD = standard deviation.

**Figure 3:**
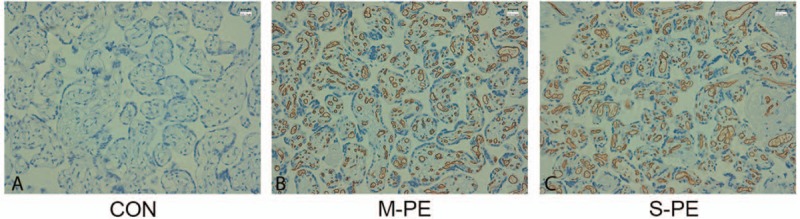
IL-8 protein expression in placental tissues determined with IHC assay. Representative IHC images of IL-8 from (A) CON, (B) M-PE, and (C) S-PE groups are shown. CON = control, IHC = immunohistochemical examination, IL-8 = interleukin-8, M-PE = mild preeclampsia, S-PE = severe preeclampsia.

Thirty placental samples from each group were randomly selected to determine IL-8 expression. Results of quantitative real-time PCR analysis showed that the relative IL-8 mRNA expression was S-PE > M-PE > control group (Fig. 2). The protein expression of IL-8 was detected on immunohistochemical (IHC) assay. As shown in the representative IHC results from each group, IL-8 was largely expressed in the cytoplasm of epithelial cells, glandular epithelium, intercellular substance of vascular endothelial cells, and perivascular interstitial cells in the placental tissues of PE patients (Fig. 3). No expression of IL-8 was detected in the control placental tissues (Fig. 3). Furthermore, 30 samples from each group were divided into 3 subgroups based on the IHC criteria for IL-8 described before. IL-8–positive ratios in control, M-PE, and S-PE were 36.7%, 83.3%, and 90%, respectively (Table 3). Chi-square test showed that IL-8–positive samples were more predominant in the 2 PE groups, but not in the control group (Table 3). Strongly positive expression of IL-8 was observed in 43.3% of the M-PE patients and in 56.7% of S-PE patients. Collectively, the above-mentioned results suggested a positive correlation of IL-8 expression with the severity of PE.

**Table 3:**
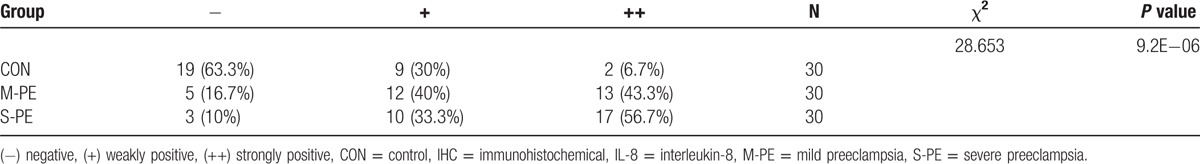
IL-8 protein expression in placental tissues determined by IHC.

### 3.4 Genotype and allele frequency of *IL-8* gene

To explore whether the genetic polymorphism of *IL-8* gene played a role in PE, we examined the distribution of 3 SNPs of *IL-8* gene (−353A/T, −251T/A, and +678T/C) and their frequencies in 160 PE and 140 control subjects. The allele frequency distributions in the healthy control (χ^2^ = 0.77, *P* = 0.38) and PE groups (χ^2^ = 3.87, *P* = 0.05) were consistent with Hardy–Weinberg equilibrium (*P* > 0.05). The frequency of *IL-8*-251A allele was 58.4% in the PE group, which was markedly higher than that in the control group (48.9%, *P* < 0.05). Allele T showed an inverse pattern (Table 4). We next examined haplotypes based on the 3 SNPs in *IL-8* gene, and found a total of 8 haplotypes. The frequencies of these haplotypes are listed in Table 5. Linkage disequilibrium analysis revealed |D′| = 0.923 for alleles −353A and −251A, 0.817 for alleles −353A and +678T, and 0.854 for alleles −251T and +678T. We noted that the higher frequency of haplotype −353A/−251A/+678T (AAT) might be associated with PE (PE vs control = 27.2% vs 21.9%, odds ratio = 1.491, 95% confidence interval = 1.005–2.213, *P* = 0.047; Table 5). Taken together, these results suggest that the AAT haplotype carriers may be more susceptible to PE.

**Table 4:**
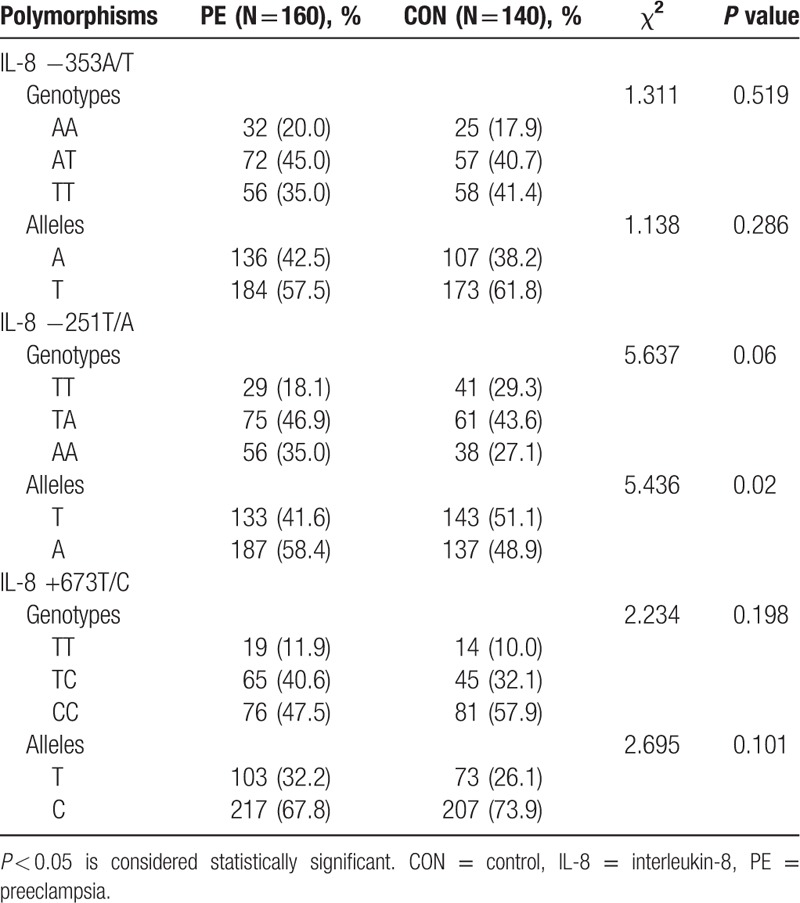
Genotype and allele frequencies of *IL-8* polymorphisms.

**Table 5:**
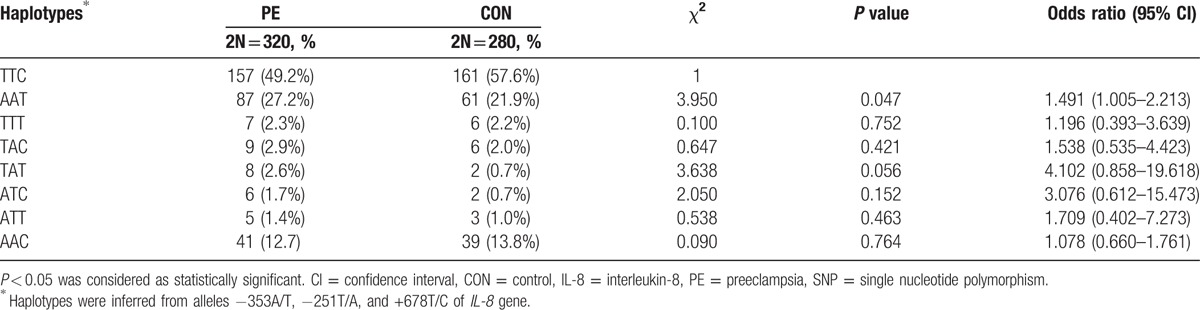
Haplotype frequencies of *IL-8* gene based on 3 SNPs.

## 4 Discussion

In the present study, we investigated the role of IL-8 in the pathogenesis of PE by comparing IL-8 expression in PE patients with discrepant clinical features (mild and severe) and healthy donors and by analyzing the genetic polymorphisms of *IL-8* gene.

Activated macrophages have been found to be present around the uterine vessels to inhibit trophoblast invasion by generating proinflammatory tumor necrosis factor-α.^[15]^ This inadequate trophoblast invasion induced by activated macrophages can be mitigated by immunoregulatory cytokine IL-10.^[16]^ Besides macrophages, activated neutrophils and natural killer cells may also provoke inflammation and lead to endothelial dysfunction.^[6]^ IL-8 is first discovered as a chemokine for neutrophils, and commonly functions through binding to its 2 CXC receptors.^[17]^ Luppi and Deloia reported a higher number of IL-8–positive peripheral blood monocytes in PE cases.^[18]^ Consistent with this previously reported study, our work also revealed a higher expression of IL-8 in serum and placenta of PE subjects. Notably, IL-8 was even higher in patients with more S-PE. There is no significant difference in serum IL-8 between pregnant women with and without cervical infection and symptoms of imminent preterm delivery.^[19]^ These results suggest a specific role of higher IL-8 level in the development of PE.

Genetic polymorphisms in *IL-8* gene have been reported to occur in human diseases; however, a majority of previous studies were focused on various cancers.^[20–23]^ Little is known about the association of genetic polymorphisms of *IL-8* gene with PE. In this study, sequence information at 3 loci (−353, −251, and +678) of *IL-8* gene was assessed to determine possible genetic polymorphisms. A previous study has shown that the *IL-8*-251A allele tends to be associated with increased IL-8 produced by lipopolysaccharide-stimulated whole blood.^[24]^ Interestingly, we found a significantly higher frequency of *IL-8*-251A allele in PE cases, which indicates that this allele may be associated with the higher expression of IL-8 in these patients. Furthermore, the occurrence frequency of haplotype −353A/−251A/+678T (AAT) in PE subjects was 27.2% as compared to 21.9% in control participants. To the best of our knowledge, our study is the first to show that pregnant women with the −353A/−251A/+678T (AAT) haplotype may be more susceptible to PE.

There are several limitations in the current study. First, our findings are based on a small sample size; further studies in a larger sample are required. Second, we have only selectively studied SNPs associated with high risk in the current study, instead of covering all SNPs potentially associated with risk. This may have introduced bias and omission in our findings, which can be resolved by including more or all SNPs in the future. Third, we did not analyze the association between the susceptibility genes with pathophysiological indices including blood biomarkers. These limitations can be hopefully overcome in future studies.

In summary, we demonstrate that PE patients have a higher expression of IL-8 in their serum and placenta, as compared to healthy controls. Moreover, a higher IL-8 expression was associated with more S-PE. The frequency of haplotype AAT was significantly higher in PE patients as compared to that in healthy controls, which suggests that AAT haplotype carriers may be at a higher risk of PE.
